# Supplementary material for: Clinical Characterization and Outcomes of Culture- and Polymerase Chain Reaction-Negative Cases of Infectious Keratitis
Source: Diagnostics (Basel). 2023 Jul 29;13(15):2528. doi: 10.3390/diagnostics13152528 (PMC10417528; doi:10.3390/diagnostics13152528)
Supplement: Supplementary file 1 [file diagnostics-13-02528-s001.zip › diagnostics-2435128-supplementary.pdf]

Supplementary Table S1. Systemic history of patients included in the study

Systemic disease	Percentage (N=167)
DMII	17.37% (29)
HTN	29.94% (50)
HLD	21.56% (36)
GERD	10.78% (18)
MRSA	1.2% (2)
Recurrent URI	4.79% (8)
Systemic Steroids	9.58% (16)
CN Palsy	2.99% (5)
Autoimmune Disease	12.57% (21)
Migraine	8.38% (14)
Heart Disease	15.57% (26)
Cognitive Impairment	1.8% (3)
Cancer	11.98% (20)
Cold Sores	10.18% (17)
Zoster	4.79% (8)
Depression	16.77% (28)
Anxiety	8.98% (15)
Coma/Intubated	1.2% (2)
Rheumatoid Arthritis	4.79% (8)
Multisystem Organ Fail	0.6% (1)
Contact Dermatitis	2.4% (4)
Tobacco Use	21.56% (36)
Substance Use	18.56% (31)
Chronic Kidney Disease	1.8% (3)
Cirrhosis	0.6% (1)
Allergies	49.7% (83)
Asthma	11.38% (19)
Hepatitis	2.99% (5)
